# Associations of Prenatal Exposure to Organophosphate Pesticide Metabolites with Gestational Age and Birth Weight

**DOI:** 10.1289/ehp.1104615

**Published:** 2012-04-05

**Authors:** Stephen A. Rauch, Joe M. Braun, Dana Boyd Barr, Antonia M. Calafat, Jane Khoury, M. Angela Montesano, Kimberly Yolton, Bruce P. Lanphear

**Affiliations:** 1 Child & Family Research Institute, BC Children’s Hospital, Vancouver, British Columbia, Canada; 2 Department of Environmental Health, Harvard University, Boston, Massachusetts, USA; 3 Rollins School of Public Health, Emory University, Atlanta, Georgia, USA; 4 National Center for Environmental Health, Centers for Disease Control and Prevention, Atlanta, Georgia, USA; 5 Department of Pediatrics, Cincinnati Children’s Hospital Medical Center, Cincinnati, Ohio, USA; 6 Simon Fraser University, Vancouver, British Columbia, Canada

**Keywords:** birth weight, DAPs, fetal growth, gestational age, OPs, organophosphate, paraoxanase, pesticide, PON, prenatal, toxicity

## Abstract

Background: Prenatal exposure to organophosphate (OP) insecticides, a widely used class of pesticides, may be associated with decreased gestational age and lower birth weight. Single nucleotide polymorphisms in paroxanase (*PON1*) enzyme genotypes may modify the relationships between OP exposure and perinatal outcomes.

Objective: We examined the relationship of prenatal OP insecticide exposure, measured using urinary dialkyl phosphate (DAP) metabolite concentrations, with gestational age and birth weight.

Methods: We measured the concentrations of six nonspecific DAP metabolites of OP insecticides in two maternal spot urine samples collected in a prospective birth cohort. We performed multivariable regression to examine associations between the sum of six DAP concentrations (ΣDAP) with gestational age and birth weight. We also examined whether these associations differed according to infant *PON1_192_* and *PON1_–108_* genotypes.

Results: Among 306 mother–infant dyads, a 10-fold increase in ΣDAP concentrations was associated with a decrease in covariate-adjusted gestational age [–0.5 weeks; 95% confidence interval (CI): –0.8, –0.1] and birth weight (–151 g; CI: –287, –16); the decrements in birth weight were attenuated after adjusting for gestational age. The relationship between ΣDAP concentrations and gestational age was stronger for white (–0.7 weeks; CI: –1.1, –0.3) than for black (–0.1 weeks; 95% CI: –0.9, 0.6) newborns. In contrast, there was a greater decrease in birth weight with increasing urinary ΣDAP concentrations for black (–188 g; CI: –395, 19) than for white (–118 g; CI: –296, 60) newborns. Decrements in birth weight and gestational age associated with ΣDAP concentrations were greatest among infants with *PON1_192QR_* and *PON_–108CT_* genotypes.

Conclusions: Prenatal urinary ΣDAP concentrations were associated with shortened gestation and reduced birth weight in this cohort, but the effects differed by race/ethnicity and *PON1_192_*_/_*_108_* genotypes.

Fifty years ago, Rachel Carson warned about the toxicity of pesticides, including organophosphate (OP) insecticides, in her book *Silent Spring* (Carson 1962). In particular, Carson warned that pesticides were toxic. Despite these warnings, there have been few attempts to study the consequences of fetal exposures to organophosphate insecticides until recently, when several epidemiologic studies using urinary pesticide metabolites have begun to raise concerns about adverse health effects of exposure (Bouchard et al. 2011; Engel et al. 2007, 2011; Eskenazi et al. 2004; Marks et al. 2010; Rauh et al. 2011). Although OP insecticide use in the United States has declined, the vast majority of people in the United States, including pregnant women and children, are routinely exposed (Bouchard et al. 2010; Woodruff et al. 2011).

The developing fetus may be especially vulnerable to OP pesticides because of its small size, rapid growth and development, and limited ability to detoxify harmful substances (Eskenazi et al. 1999). Animal studies suggest that prenatal OP insecticide exposure at levels higher than those typically encountered by humans reduces fetal growth (Spyker and Avery 1977; Srivastava and Raizada 1996). However, results from several epidemiologic studies that sampled from different racial and ethnic populations have been inconclusive. Among African-American and Dominican mothers living in New York City, Whyatt et al. (2004) reported an inverse association between cord blood concentrations of chlorpyrifos, an OP insecticide, with birth length and birth weight. In a cohort of primarily Mexican-American mothers, Eskenazi et al. (2004) reported an association between prenatal urinary dialkyl phosphate (DAP) concentrations, which are metabolites of OP insecticides, and shortened gestation, but not gestation-adjusted birth weight.

Paraoxonase (PON) is an enzyme that detoxifies several OP pesticides, including chlorpyrifos, parathion, and diazinon (Costa et al. 2003). Several polymorphisms in the *PON1* gene affect the function, production, and efficiency of PON in the body; recent studies have focused on two alleles: *PON1_192_* and *PON1_–108_* (Costa et al. 2003; Deakin et al. 2003). Briefly, a change in the promoter region of *PON1_–108_* from the C allele to the T allele causes decreased PON production, and a change in the coding region of *PON1_192_* from the R to the Q allele decreases PON’s efficiency at detoxifying certain pesticides (Costa et al. 2003). In epidemiologic studies, results have been inconclusive: Harley et al. (2011) found larger DAP-associated reductions in birth weight and gestational age in infants with *PON1_–108TT_* genotype (vs. *PON1_–108CC_*); for *PON1_192_*, they found DAP-associated reductions in gestational age for QQ infants, and weaker associations for the RR or QR groups. However, Wolff et al. (2007) identified *PON1_192RR_* as the more susceptible group, reporting stronger negative associations between diethyl alkyl phosphate (DEAP) metabolites and birth weight, and between dimethyl alkyl phosphate (DMAP) metabolites and birth length, in mothers with the *PON1_192RR_* genotype (compared with those with QQ genotype). Berkowitz et al. (2004) found no overall relationship between a urinary chlorpyrifos metabolite (3,5,6-trichloropyridinol) and gestational age, birth weight, and birth length, but they found an inverse association with head circumference when stratifying by tertiles of maternal paraoxonase activity.

In the United States, black children are more likely than white children to be born preterm (gestation < 37 weeks) and to be low birth weight (< 2,500 g), both of which place newborns at risk for adverse health outcomes (Wise et al. 1985). Racial differences in gestational age and birth weight may be a result of adverse environmental exposures, genetics, socioeconomic status, and/or other unidentified factors. Several studies in the United States have found that black individuals are often exposed to higher levels of environmental toxicants than white individuals, but it is unclear whether these exposures contribute to racial differences in gestational age or birth weight (Gee and Payne-Sturges 2004). There is also evidence that *PON1* distributions differ across racial groups (Davis et al. 2009).

The purpose of this study was to examine the relationship of prenatal OP insecticide exposure, measured using urinary concentrations of six DAP metabolites, with gestational age and birth weight in a birth cohort from Cincinnati, Ohio. We hypothesized that gestational OP exposure would be associated with shorter gestation and lower birth weight. We also hypothesized that the relationship between urinary OP exposure and birth weight/gestational age would be greatest among infants with *PON1_192QQ_* or *PON1_–108TT_* genotypes.

## Methods

*Study sample.* We used data collected from mothers and newborns enrolled in the Health Outcomes and Measures of the Environment (HOME) Study, an ongoing prospective birth cohort. Subject recruitment and interviews for the HOME Study have been described elsewhere (Braun et al. 2010). Briefly, we identified and contacted women attending seven prenatal clinics in the Cincinnati metropolitan area between March 2003 and January 2006. Eligibility criteria included being at least 18 years of age; living in a home built before 1978; ≤ 19 weeks of gestation; being HIV negative; living within five surrounding counties; and not receiving thyroid or seizure medications, or chemotherapy or radiation treatments. Institutional review boards of all involved research institutions, hospitals, and laboratories approved the study protocol. All mothers gave written informed consent for themselves and their children before enrolling. Of 1,263 eligible women, 468 enrolled in the study. Sixty-seven women dropped out of the study before delivery. The remaining 401 women gave birth to 389 live singleton babies, as well as 9 sets of twins and 3 still-born infants. Of those delivering singletons, 344 women provided urine at both 16 and 26 weeks gestation; they make up our primary study sample.

*Measurement of pesticide exposure.* Participants provided blood and spot urine samples at 16 and 26 weeks gestation, and within 24 hr of delivery. Urine was collected in polypropylene containers and stored at –20°C until shipment to the Centers for Disease Control and Prevention (CDC) for analysis. We measured six urinary DAPs—dimethylphosphate (DMP), dimethylthiophosphate (DMTP), dimethyldithiophosphate (DMDTP), diethylphosphate (DEP), diethylthiophosphate (DETP), and diethyldithiophosphate (DEDTP), which are common metabolites of about 75% of the OP insecticides used in the United States—using a modification of the analytical method of Bravo et al. (2004) that employs gas chromatography-tandem mass spectrometry with isotope dilution calibration.

Quality control (QC) materials, prepared at CDC from spiked pooled urine, were analyzed with standards, blanks, and study samples. The QC concentrations were evaluated using standard statistical probability rules (Caudill et al. 2008). The limits of detection (LODs) varied depending on the metabolite and ranged from 0.2 μg/L for DMTP to 0.6 μg/L for DMP. For samples reporting nonzero concentrations below the LOD, we used the reported value. We imputed DAP concentrations that were reported as zero by choosing a random number between zero and the lowest nonzero value for that metabolite.

We converted the metabolite concentrations from micrograms per liter to their molar concentrations (nanomoles per liter) and summed them to obtain overall concentrations of diethyl alkyl phosphates (ΣDEAPs: DEP, DETP, and DEDTP), dimethyl alkyl phosphates (ΣDMAPs: DMP, DMTP, DMDTP), and of all six metabolites (ΣDAPs). We used creatinine as a marker of urine dilution and obtained creatinine-standardized DAP concentrations by dividing metabolite concentrations by creatinine concentration (Larsen 1972). To avoid confounding due to metabolic changes that occur around the time of delivery, we confined our analysis to the 16- and 26-week results, and restricted the analysis to mothers with data from both time points. Because metabolite concentrations varied widely between time points, we averaged the concentrations of each metabolite across the two samples. Because the distribution of the averaged DAP concentrations was right-skewed, we log_10_-transformed the averages to achieve a more normal distribution.

*Outcome and covariate measures.* We abstracted birth weight from medical records, and calculated gestational age from the mother’s self-reported date of last menstrual period. When this information was not available, we used the results of an ultrasound (*n* = 7) or a Ballard examination performed just after delivery (*n* = 3) to determine gestational age. Demographic factors (i.e., maternal age, education, race/ethnicity, parity, income, marital status) were obtained from subject interviews. We also characterized infant size using race-specific birth weight for gestational age *z*-scores (e.g., birth weight standardized for gestational age) to determine whether infants’ growth was restricted relative to their length of gestation (Oken et al. 2003).

We measured serum cotinine, a metabolite of nicotine that has been validated as a biomarker of secondhand and active tobacco smoke exposure (Benowitz et al. 2009; DeLorenze et al. 2002), and whole blood lead concentrations from blood samples taken at the same time as the spot urine samples. As with the urinary DAP concentrations, we averaged serum cotinine and blood lead concentrations across the 16- and 26-week samples and log_10_-transformed the results. Several women were missing lead or cotinine measurements for one or both time points; if a lead or cotinine measurement was missing for one time point, we used the value for the other time point instead of the average. Participants who were missing blood lead or serum cotinine concentrations from both time points (*n* = 18) were excluded from the analysis. Blood lead and serum cotinine were quantified using previously described methods at the CDC laboratories (Bernert et al. 2000; CDC 2003).

*PON genotyping data.* DNA was extracted from frozen archived cord blood using the 5PRIME PerfectPure DNA blood kit (5PRIME, Gaithersburg, MD, USA). Genotyping was conducted at the Cincinnati Children’s Hospital Medical Center DNA sequencing and genotyping core facility. We used the Applied Biosystems predesigned TaqMan assays (Applied Biosystems, Carlsbad, CA, USA) for rs662 (192Q/R) and rs705379 (–108C/T) with 15 ng of genomic DNA. The protocol was as per the manufacturer’s recommendations in 384 well format, including 16 blanks and 4 sets of 2 controls for QC purposes. Assay plates mixing reagents and DNA were prepared using a TECAN Evo200 robotic laboratory workstation (TECAN, Durham, NC, USA) and amplified in an ABI 9700 thermocycler (Applied Biosystems). TaqMan results were read on an ABI 7900HT real time PCR system and exported in Excel format using the ABI SDS 2.4 software (Applied Biosystems). Genotype data was available for 312 of 344 (90.7%) of infants, with 300 (87.2%) having identifiable SNPs at both *PON1* sites.

*Statistical analysis.* For multivariable analysis, we used PROC GLM in SAS version 9.1 (SAS Institute Inc., Cary, NC, USA). We began with an *a priori* set of covariates that have been consistently associated with gestational age and birth weight: maternal age, education, race/ethnicity, and marital status; household income; gestational age at initiation of prenatal care; and parity. We also considered the following covariates: depressive symptoms (measured by the Beck Depression Inventory-II) (Beck et al. 1996), IQ, insurance status, area of residence, maternal body mass index, alcohol use during pregnancy, child sex, tobacco exposure (assessed using serum cotinine), and blood lead concentrations. Any of these covariates showing univariate associations (*p* < 0.2) with both DAP concentrations and birth outcomes were included in our multivariable analyses. Only blood lead and serum cotinine concentrations fit these criteria. Our final model included maternal age (continuous), race/ethnicity (black or white), and marital status (married or not married), household income (contiuous), parity (0, 1, or ≥ 2), and blood lead and serum cotinine concentrations (log_10_-transformed). We did not include gestational age in the primary birth weight analysis because of its potentially being on the causal pathway. However, for comparison with prior results in other cohorts, we constructed gestational age-adjusted models in a sensitivity analysis.

We assessed the form of the exposure–response relationship using locally weighted regression (LOESS) plots of log-transformed concentrations and outcomes. Because a straight line fit inside the 95% confidence bands, we concluded that it was appropriate to use a linear term to describe the relationship.

Based on our preliminary analyses, we observed that the pattern for associations of DAP concentrations and birth outcomes varied by race/ethnicity. Because almost all mothers identified themselves as either black or white (the “other” group contained only 15 mothers), we restricted our analyses to black and white mothers. We then ran race-stratified models to obtain effect estimates, and formally assessed interactions in separate models using cross-product terms between race and urinary OP pesticide metabolite concentrations, using *p* < 0.1 as the criteria for a statistically significant interaction.

To examine gene–environment interactions between *PON1* genotype and OP pesticides, we ran several PON-stratified analyses, stratifying by *PON1_192_* or *PON1_–108_*. We also formally assessed interactions using dummy variables and interaction terms. Because race appeared to be an effect modifier and *PON1* genotype can vary across racial groups, we also ran the *PON1*-stratified analyses separately for black and white mothers whenever possible given sample sizes.

We also performed several sensitivity analyses. First, we examined whether mothers’ pregnancy medical conditions might confound the relationship between urinary DAP concentrations and birth outcomes. These included abruptio placenta, placenta previa, chorioamnionitis, preeclampsia, and pregnancy-induced hypertension. Next, we examined whether relationships between DAP concentrations and birth weight were evident for only term newborns (≥ 37 weeks) (Wilcox 2006). We considered child sex as an effect modifier and assessed interactions with cross-product terms. Next, we assessed whether different methods of creatinine adjustment altered our results. We used DAP concentrations as our exposure variable without creatinine standardization or adjustment, and also ran a model with log_10_-transformed mean urinary creatinine concentrations as a covariate, as suggested by Barr et al. (2005). We also examined whether dilute urine samples changed the pattern of our results by excluding women who had at least one sample with creatinine < 20 mg/dL. Finally, to assess the effects of different time periods of sample collection, we ran separate models using the DAP concentrations in samples taken at the 16-week and 26-week visits.

## Results

Mothers participating in the study were predominantly white, 25–35 years old, married, and primiparous (Table 1). Household income and education varied among the women, with the greatest proportion falling in the $55,000–$85,000 range, with either a high school diploma or bachelor’s degree. Relatively few infants were born preterm (*n* = 31, 9.0%) or were low birth weight (< 2,500 g, *n* = 20, 5.8%).

**Table 1:** Demographic characteristics of HOME study participants: Cincinnati, Ohio, 2003–2006 (n = 344).^a^

n (%)	Birth weight (g) (mean ± SD)	Gestational age (weeks) (mean ± SD)	ΣDAPs (nmol/L) Median (25th–75th)
All participants		344 (100)		3,383 ± 622		39.0 ± 1.7		81.3 (41.7–220.0)
Age (years)								
Under 25		72 (21.4)		3,055 ± 619		38.6 ± 2.2		108.1 (55.0–244.2)
25–29.9		96 (28.6)		3,508 ± 576		39.3 ± 1.5		77.9 (41.7–220.0)
30–34.9		111 (33.0)		3,476 ± 573		39.1 ± 1.6		66.7 (39.3–169.8)
35 or Older		57 (17.0)		3,392 ± 682		38.7 ± 1.7		93.7 (46.9–232.2)
Race/ethnicity								
Black		97 (28.4)		3,128 ± 539		38.6 ± 1.8		126.5 (44.8–319)
White		229 (67.2)		3,516 ± 602		39.2 ± 1.5		75.2 (41.3–192.3)
Other		15 (4.4)		3,249 ± 648		38.9 ± 2.3		142.6 (73.4–231.6)
Household income								
< $22,500		64 (19.2)		3,121 ± 467		38.9 ± 1.5		97.5 (41.7–254.2)
$22,500–$45,000		86 (25.7)		3,432 ± 658		38.9 ± 1.7		101.8 (39.7–245.7)
$55,000–$85,000		117 (35.0)		3,515 ± 634		39.1 ± 1.8		75.0 (36.5–169.8)
> $85,000		67 (20.1)		3,410 ± 561		39.2 ± 1.7		104.2 (52.5–213.7)
Education								
Less than high school		32 (9.4)		3,130 ± 533		38.8 ± 1.6		92.4 (41.4–216.5)
High school diploma		129 (37.8)		3,316 ± 581		39.0 ± 1.7		76.1 (36.2–192.7)
Bachelor’s degree		102 (29.9)		3,532 ± 606		39.2 ± 1.6		85.0 (44.1–243.6)
Graduate degree		78 (22.9)		3,451 ± 652		39.0 ± 1.8		91.8 (47.6–219.7)
Marital status								
Married		233 (68.3)		3,477 ± 616		39.1 ± 1.7		80.7 (43.6–219.6)
Not married		108 (31.7)		3,215 ± 563		38.9 ± 1.7		90.6 (40.4–239.9)
Parity								
0		155 (45.2)		3,342 ± 607		39.3 ± 1.6		99.1 (46.3–234.1)
1		109 (31.8)		3,451 ± 587		39.0 ± 1.5		76.1 (41.9–192.7)
≥ 2		79 (23.0)		3,369 ± 697		38.4 ± 2.1		66.0 (32.6–192.3)
Child sex								
Female		181 (52.6)		3,290 ± 572		39.0 ± 1.7		76.1 (39.2–207.4)
Male		163 (47.4)		3,487 ± 660		39.0 ± 1.7		90.2 (46.3–232.2)
25th and 75th are percentiles. aStudy population included women who provided spot urine samples at the 16-week and 26-week visits.								

All mothers had detectable levels of at least one DMAP metabolite, and at least one DEAP metabolite was detected in 92.7% of women. The ΣDAP metabolites median was 81.3 nmol/L (IQR = 42–220) (Table 1). Concentrations were higher at 16 weeks than at 26 weeks; however, creatinine-standardized DAP concentrations were similar at both time points. The two creatinine-standardized measurements were weakly correlated (*r* = 0.22). ΣDMAP metabolite concentrations (median = 56.9 nmol/L; IQR = 26–185) were considerably higher than those of ΣDEAP metabolites (median = 17.7 nmol/L; IQR = 8–37). The median blood lead concentration was 0.66 µg/dL (IQR = 0.51–0.87), and the median serum cotinine level was 0.027 ng/mL (IQR = 0.01–0.18). In the final models, we dropped one mother because of a missing creatinine value, 22 because of missing covariate values, and 15 in the “other race” category, giving us a final sample size of 306.

*PON1* genotype was also associated with maternal race/ethnicity in the study sample. Infants born to white mothers were more likely to have *PON1_192QQ_* (51.4% vs. 12.7%) and *PON1_–108_*_CT_ genotypes (45.7% vs. 20.0%) than infants born to black mothers. Infants with black mothers were more likely to have *PON1_192_*_RR_ (44.3% vs. 10.4%) and *PON1_–108_*_CC_ (76.3% vs. 29.8%) genotypes (all chi-square *p*-values < 0.001).

In multivariable analysis, a 10-fold increase in ΣDAP concentrations, which corresponds roughly to a shift from the 15th (29.5 nmol/L) to the 85th percentile (318.0 nmol/L) of the distribution, was associated with a 0.5-week decrease in gestational age [95% confidence interval (CI): –0.8, –0.1] (Table 2). Associations with ΣDEAP and ΣDMAP concentrations were weaker, but ΣDMAP concentrations were associated with a 0.4-week decrease in gestational length (95% CI: –0.7, 0.0). Birth weight was also inversely associated with ΣDAP concentrations (–151 g, 95% CI: –287, –16) and ΣDMAP concentrations (–124 g, 95% CI: –245, –2), but not ΣDEAP concentrations. The relationship between ΣDAP concentrations and birth weight was attenuated after adjustment for gestational age (–40 g; 95% CI: –146, 65).

**Table 2:** Mean change in gestational length and birth weight associated with a 10-fold increase in average urinary DAP concentrations (nmol/L) among white and black mothers.

OP Metabolite^a^		Length of gestation (weeks)^b^						Birth weight (g)^b^						Birth weight, adjusted for gestational age (g)^c^				
		β (95% CI)		p-value		p_interaction_^d^		β (95% CI)		p-value		p_interaction_^d^		β (95% CI)		p-value		p_interaction_^d^
All mothers (n = 306)																		
ΣDAP		–0.5 (–0.8, –0.1)		0.01		NA		–151 (–287, –16)		0.03		NA		–40 (–146, 65)		0.45		NA
ΣDEAP		–0.2 (–0.5, 0.1)		0.14				–65 (–180, 51)		0.27				–9 (–99, 80)		0.83		
ΣDMAP		–0.4 (–0.7, 0.0)		0.03				–124 (–245, –2)		0.05				–38 (–133, 56)		0.43		
Black mothers (n = 93)																		
ΣDAP		–0.1 (–0.9, 0.6)		0.69		0.10		–188 (–395, 19)		0.07		0.46		–158 (–297, –18)		0.03		0.02
ΣDEAP		–0.1 (–0.8, 0.5)		0.64		0.47		–162 (–340, 16)		0.07		0.39		–131 (–251, –11)		0.03		0.08
ΣDMAP		0.0 (–0.7, 0.6)		0.97		0.09		–142 (–333, 50)		0.15		0.46		–139 (–267, –10)		0.03		0.02
White mothers (n = 213)																		
ΣDAP		–0.7 (–1.1, –0.3)		< 0.01		NA		–118 (–296, 60)		0.19		NA		60 (–84, 204)		0.41		NA
ΣDEAP		–0.3 (–0.7, 0.0)		0.09				–39 (–189, 111)		0.61				41 (–78, 160)		0.5		
ΣDMAP		–0.6 (–0.9, –0.2)		< 0.01				–96 (–254, 62)		0.23				49 (–78, 177)		0.45		
NA, not applicable. Excludes mothers in the “other race” category (n = 15) and with missing covariate information (n = 23). A 10-fold increase in total DAP concentrations (i.e., a 1-unit increase in log10‑transformed concentrations) corresponds roughly to an increase from the 15th (29.5 nmol/L) to the 85th percentile (318.0 nmol/L) of the distribution. aMetabolites are log10-transformed and creatinine-standardized. bAdjusted for mother’s age, household income, marital status, parity category, and log10-transformed blood lead and log10-transformed cotinine. Models including black and white mothers are also adjusted for maternal race. cAdditional adjustment for gestational age. dp-Value for race × exposure interaction term from separate cross-product models.																		

In race-stratified models, ΣDAP concentrations were associated with shortened gestation in white newborns, but not in black newborns (Table 2). The interaction between ΣDAP concentrations and black race/ethnicity in separate cross-product models was significant at *p* = 0.10. In contrast, ΣDAP concentrations were associated with a larger decrease in birth weight among black newborns than in white newborns, although the interaction was not statistically significant (*p* = 0.46). Once we adjusted for gestational age, however, we observed a slightly weaker but statistically significant inverse association between ΣDAP concentrations and birth weight for black newborns, and the interaction for ΣDAP concentrations and birth weight by race/ethnicity became statistically significant (*p* = 0.02). We also found a nonsignificant inverse relationship between urinary OP metabolite concentrations and birth weight for gestational age (–0.18 *z*-score units; 95% CI: –0.41, 0.06). The relationship was stronger among blacks (–0.40; 95% CI: –0.73, –0.08) than whites (–0.02; 95% CI: –0.33, 0.30).

In *PON1*-stratified models, we observed the strongest associations between ΣDAP concentrations and birth weight and gestational age in the heterozygous groups: *PON1_192QR_* and *PON1_–108CT_* [see Supplemental Material, Table 1 and Figures 1 and 2 (http://dx.doi.org/10.1289/ehp.1104615)]. Several interaction terms between *PON1* genotypes and ΣDAP concentrations were statistically significant. Looking at models stratified by race and *PON1* status, numbers of observations in many of the categories were too small to produce stable point estimates. However, in almost all cases we observed the strongest associations between DAP metabolites and birth outcomes appearing in the heterozygous groups. We found no evidence for main effects of *PON1* genotype on either birth weight or length of gestation (data not shown).

*Sensitivity analyses.* Medical conditions were associated with birth outcomes and DAP urinary concentrations (Table 3). Mothers who had abruptio placenta (*n* = 9), placenta previa (*n* = 1), chorioamnionitis (*n* = 1), pre-eclampsia (*n* = 15), or pregnancy-induced hypertension (*n* = 9) had shorter gestations and lower-birth-weight newborns than the rest of the study population (data not shown). These mothers also had higher median DAP urinary concentrations than mothers without the conditions (Table 3), although we did not perform formal statistical tests because of small sample sizes. Generally, the magnitude of association between ΣDAP concentrations and gestational age was similar when we excluded observations based on various combinations of the medical conditions. The point estimates for birth weight ranged from –168 g (95% CI: –307, –28) when women with hypertension were excluded to –111 g (CI: –248, 26) when women with placental conditions, chorioamnionitis, or hypertension were all excluded (all estimates reported for sensitivity analyses are adjusted for the same covariates as in the primary analysis, unless otherwise noted).

**Table 3:** Median ΣDAP concentration in mothers with and without selected medical conditions and mean change in gestational length and birth weight associated with a 10-fold increase in average urinary ΣDAP concentrations after exclusion based on medical conditions.

Median ΣDAP concentration (nmol/L) (25th–75th)			Gestational age (weeks)^a^			Birth weight (g)^a^			Birth weight, adjusted for gestational age (g)^b^		
Condition	n	With condition	Without condition	β (95% CI)	p-value	β (95% CI)	p-value	β (95% CI)	p-value
None		306		NA		77.9 (41.6–207.4)		–0.5 (–0.8, –0.1)		0.01		–151 (–287, –16)		0.03		–40 (–146, 65)		0.45
Placental/chorio		296		133.3 (82.7–192.7)		76.1 (41.2–210.6)		–0.3 (–0.6, 0.0)		0.08		–115 (–245, 15)		0.08		–48 (–154, 59)		0.38
Hypertensive		288		110.3 (63.8–190.9)		76.2 (41.4–210.5)		–0.6 (–0.9, –0.2)		< 0.01		–168 (–307, –28)		0.02		–30 (–143, 83)		0.60
Placental/chorio and hypertensive		282		128.3 (64.8–237.1)		75.7 (41.1–207.4)		–0.3 (–0.7, 0.0)		0.06		–111 (–248, 26)		0.11		–33 (–145, 79)		0.56
NA, not applicable. 25th and 75th are percentiles. Excludes 15 mothers in “other race” category and 23 with missing covariate information. “Placental/chorio” (n = 10) includes 8 mothers with abruptio placenta, 1 with abruption placenta and placenta previa, and 1 with chorioamnionitis. “Hypertensive” (n = 18) includes 9 mothers with preeclampsia, 3 mothers with pregnancy-induced hypertension, and 6 mothers with preeclampsia and pregnancy-induced hypertension. Four mothers had preeclampsia and hypertensive conditions. aAdjusted for mother’s age, race, household income, marital status, parity category, and log10-transformed blood lead and log10-transformed cotinine. bAdditional adjustment for gestational age.																		

Restricting analyses to full-term births (≥ 37 weeks gestation, *n* = 282) attenuated the effect estimates for a 10-fold increase in ΣDAP concentrations for both gestational age (–0.2 weeks, 95% CI: –0.4, 0.0) and birth weight (–88 g, 95% CI: –213, 37), but the patterns were consistent with our primary results. Stratifying full-term births by race produced results that were similar to those for preterm and term births combined: Associations with birth weight were stronger among black newborns than white newborns, whereas estimated effects for gestational age were stronger in white newborns than black newborns. We did not find any suggestion of an interaction between child sex and ΣDAP concentrations for either gestational age or birth weight (*p* > 0.3).

Using non-creatinine-standardized ΣDAP concentrations modestly attenuated the observed estimates: In the full group, the gestational age coefficient was –0.3 weeks (95% CI: –0.7, 0.0) and the birth weight coefficient was –100 g (95% CI: –232, 32). Adjusted coefficients in all secondary analyses were closer to the null without creatinine adjustment, but the pattern with respect to race remained the same (data not shown). Likewise, excluding women with urinary creatinine values < 20 mg/dL (*n* = 37) did not change the pattern of results (data not shown). Finally, associations obtained when using ΣDAP concentrations from individual spot urine collections were slightly attenuated compared with the average of the 16- and 26-week collections, for both the 16-week (gestational age: β = –0.2 weeks, 95% CI: –0.6, 0.1; birth weight: β = –110g, 95% CI: –221, 1) and 26-week samples (gestational age: β = –0.3 weeks, 95% CI: –0.6, 0.0; birth weight: β = –88g, 95% CI: –208, 33).

## Discussion

We found that maternal exposure to OP insecticides, assessed by measuring urinary OP pesticide metabolites, was inversely associated with gestational age and birth weight. The estimated effects of OP exposure differed by race: Gestational age was inversely associated with DAP concentrations in white, but not black mothers. In contrast, we observed a stronger inverse association between maternal DAP concentrations and birth weight among black newborns than white newborns. The same pattern persisted when we restricted the analysis to full-term newborns, although the associations were attenuated. Although we chose not to adjust for gestational age in the primary analysis because it is may be on the causal pathway, our results in white mothers are consistent with those of Eskenazi et al. (2004), who found that higher urinary ΣDMAP concentrations were associated with shortened gestation and small increases in gestational age–adjusted birth weight in a primarily Latina cohort. The results for the association of OP insecticides and birth weight for gestational age *z*-scores closely mirrored our results for birth weight adjusted for gestational age. Again, we found an association for black, but not white, infants. In addition, at 39 weeks gestation (the study population mean) the estimated *z*-score decrease of 0.4 associated with a 10-fold increase in ΣDAPs corresponds to a decrease of about 160 g (Oken et al. 2003). Although others have reported effect modification by child sex (Marks et al. 2010), we found no evidence of an interaction. It is possible that different levels of exposure could affect the results; however, we observed ΣDAP concentrations (median = 81.3 nmol/L) comparable to the urban population in the Mount Sinai birth cohort in New York City (median = 75.9 nmol/L) (Wolff et al. 2007) and lower than those in the agricultural population of the CHAMACOS cohort (median = 136 nmol/L) (Eskenazi et al. 2004). The Columbia University birth cohort used serum chlorpyrifos to categorize OP exposure, and was not directly comparable (Whyatt et al. 2004).

It is unclear why OP insecticides would be associated with shorter gestation in white newborns, but not black newborns. Although black mothers had higher levels of urinary OP pesticide metabolites than white mothers, these metabolites are a nonspecific measure of exposure to various OP insecticides; different OP pesticides may act through a variety of mechanisms, some affecting length of gestation and others affecting fetal growth. Blacks and whites might differentially be exposed to these different formulations of pesticides. Maternal race/ethnicity may also be acting as a proxy for a combination of conditions—socioeconomic factors, genetic susceptibility, psychological stressors, and environmental toxicants—only some of which were measured in our study. We examined several possible confounders, including income, education, maternal depressive symptoms, maternal IQ, insurance status, area of residence, prenatal care, *PON1* genotype, and gestational exposure to alcohol, lead, and tobacco; however there may be other factors we did not measure. Like Davis et al. (2009), we found racial differences in the distribution of *PON1* genotypes; however, the race/ethnicity interactions were not affected when we adjusted for *PON1* genotype (data not shown).

Excluding mothers with specific medical conditions, especially those who had placental abnormalities or chorioamnionitis, attenuated the association of urinary DAP concentrations and perinatal outcomes. Excluding women with hypertensive conditions, which were more common in black mothers than in white mothers, strengthened the associations to a negligible extent.

For both *PON1* polymorphisms we examined, the heterozygous groups (*PON1_192QR_* and *PON1_–108CT_*) generally showed the largest estimated decreases in birth weight and gestational length associated with log_10_-transformed ΣDAP concentrations. These results were unexpected, but > 200 polymorphisms of *PON1* have been identified, and it may be unreasonable to expect two *PON1* polymorphisms to explain the amount of PON produced or its efficiency in detoxifying certain chemicals (Costa et al. 2005, 2011). Several studies looking for OP–*PON1* interactions have measured not only genotype, but PON enzyme activity as well (Harley et al. 2011; Wolff et al. 2007). We did not measure serum levels of PON and other OP-detoxifying enzymes, or maternal *PON1* genotype, which may be more relevant during gestation and fetal development. It is also worth noting that studies on *PON1* have focused on pesticides such as chlorpyrifos, diazinon, and parathion, which metabolize into DEP and DETP, whereas other OPs such as malathion, azinphos methyl, and dimethoate metabolize into DMP and DMTP (Maroni et al. 2000). DMAP metabolites were much more abundant in our study samples, and their associations were typically stronger than the DEAP metabolite associations. It is possible that *PON1* polymorphisms act differently on DEAP- and DMAP-producing OPs.

This study has several strengths and limitations, primarily related to using urinary DAP metabolites as a marker of OP insecticide exposure. Urinary DAP measurements reflect exposure to nontoxic preformed metabolites as well as the parent (toxic) OP pesticides, particularly for dietary exposures (Lu et al. 2005). Urinary DAPs have a half-life < 24 hr in humans, so their urinary concentrations reflect only recent exposure (Barr and Angerer 2006). The combination of the short elimination half-life and the episodic nature of exposures to OP pesticides results in a high degree of variability in urinary DAP concentrations. We were also unable to rule out the possibility that differences in DAP concentrations partially reflect individual variation in metabolism. One strength of this study, however, is that we were able to account for some of the temporal variation in urinary DAP concentrations by combining the concentrations of two samples taken months apart. Although there may still have been some degree of exposure misclassification, it was most likely nondifferential, and would tend to bias our results towards the null. Another limitation is that, unlike serum measures of the OP pesticide, which are often undetectable, urinary DAP concentrations cannot quantify exposure to a particular parent pesticide, although the class of metabolites (DEAP vs. DMAP) can suggest exposure to certain groups of OP pesticides. The ability to identify specific OP exposures would improve etiological studies because OPs differ widely in their uses and relative toxicity. Similarly, we were unable to pinpoint the dominant sources of exposure to OP pesticides. Diet and home pesticide use have been identified as important routes of exposure in nonagricultural populations, although Lu et al. (2008) found that switching children from conventional to organic diets for several days reduced urinary OP metabolites to levels near or below the limit of detection, suggesting that diet was the primary source of exposure in that study population. Even if we were able to isolate a particular pesticide, it would be difficult to connect it to a particular route of exposure, as many different pesticides are used in agriculture, as well as in and around the home.

The reliance on self-reported dates of last menstrual period to determine gestational age represents another limitation (Lynch and Zhang 2007). This method is imprecise; however, there is no reason to suspect differential misclassification of gestational age with respect to OP exposure. In addition, it was recorded more consistently (97% of mothers) than was ultrasound (8%). Finally, information on PON enzyme activity or maternal *PON1* status might shed more light on PON-mediated susceptibility than infant *PON1* genotypes alone. Other strengths of this study are that we presented data from a racially and demographically mixed birth cohort in a sample, intentionally enriched with black women, from the Cincinnati metropolitan area. We also controlled for a wide range of confounders, including continuous measures of lead and tobacco exposure, and pregnancy medical complications, and our findings were robust to several sensitivity analyses.

## Conclusion

Prenatal OP insecticide exposure was associated with shorter gestation and lower birth weight in this cohort. The magnitude of these associations differed according to maternal race and infant *PON1* genotype status. Although the prior literature on gestational OP exposure and birth outcomes is inconclusive, several studies have observed associations between adverse health outcomes in children and maternal exposure to OP insecticides during gestation, including abnormal reflexes (Engel et al. 2007), reduced cognitive abilities (Bouchard et al. 2011; Engel et al. 2011; Rauh et al. 2011), and ADHD and attention problems (Bouchard et al. 2010; Marks et al. 2010). Although OP insecticide use in the United States has declined in recent years (Grube et al. 2011), exposures to them remain widespread. These results are not definitive, but they serve to remind us about Rachel Carson’s warnings about the use of chemicals of uncertain toxicity, including OP insecticides.

## Supplemental Material

(160 KB) PDF
