# Supplementary material for: Associations of Prenatal Exposure to Organophosphate Pesticide Metabolites with Gestational Age and Birth Weight
Source: Environ Health Perspect. 2012 Apr 5;120(7):1055–60. doi: 10.1289/ehp.1104615 (PMC3404666; doi:10.1289/ehp.1104615)
Supplement: (160 KB) PDF [file ehp.1104615.s001.pdf]

Supplemental Material

Associations of Prenatal Exposure to Organophosphate Pesticide Metabolites with Gestational Age and Birthweight

Stephen A. Rauch, Joe M. Braun, Dana Boyd Barr, Antonia M. Calafat,
Jane Khoury, M. Angela Montesano, Kimberly Yolton, Bruce P. Lanphear

Supplemental Material, Table 1. Mean change in gestational length and birthweight associated with a 10-fold increase in average urinary ΣDAP concentrations, stratified by *PON1* genotypes, and p-values for DAP x *PON1* interactions.

	N	Length of gestation (weeks)[a]			Birthweight (g)[a]		
		β (95% CI)	p-value	$p_{interaction}$[b]	β (95% CI)	p-value	$p_{interaction}$[b]
All Mothers							
Stratified by $PON1_{192}$ Genotype:							
RR (ref)	55	-0.3 (-1.2, 0.5)	0.44	N/A	-71 (-384, 242)	0.65	N/A
QR	107	-0.9 (-1.6, -0.3)	<0.01	0.04**	-454 (-707, -201)	<0.01	0.02**
QQ	111	-0.5 (-1.1, 0.0)	0.07	0.09*	-2 (-231, 228)	0.99	0.76
Stratified by $PON1_{-108}$ Genotype							
CC	118	-0.3 (-0.9, 0.3)	0.32	N/A	-119 (-340, 103)	0.29	N/A
CT	106	-1.0 (-1.6, -0.4)	<0.01	0.04**	-299 (-520, -78)	0.01	0.15
TT	46	-0.1 (-1.0, 0.8)	0.87	0.31	85 (-361, 530)	0.7	0.12
Black Mothers							
Stratified by $PON1_{192}$ Genotype:							
RR (ref)	28	0.3 (-1.3, 1.8)	0.74	N/A	305 (-215, 826)	0.23	N/A
QR	32	-0.4 (-2.2, 1.3)	0.61	0.47	-551 (-1058, -44)	0.03	0.05**
QQ	7	NA[c]					
Stratified by $PON1_{-108}$ Genotype:							
CC	52	0.1 (-1.0, 1.2)	0.87	N/A	-7 (-337, 323)	0.97	N/A
CT	14	0.0 (-1.5, 1.5)	0.98	0.97	-609 (-1174, -45)	0.04	0.37
TT	2	NA[c]					
White Mothers							
Stratified by $PON1_{192}$ Genotype:							
RR (ref)	22	-0.4 (-2, 1.1)	0.55	N/A	38 (-693, 769)	0.91	N/A
QR	69	-0.7 (-1.3, -0.1)	0.03	0.05**	-344 (-663, -25)	0.04	0.09*
QQ	96	-0.5 (-1.0, 0.0)	0.07	0.06*	66 (-186, 320)	0.6	0.58
Stratified by $PON1_{-108}$ Genotype:							
CC	58	-0.6 (-1.2, 0.1)	0.08	N/A	-194 (-551, 162)	0.28	N/A
CT	84	-0.8 (-1.3, -0.2)	<0.01	0.64	-178 (-438, 81)	0.17	0.75
TT	42	-0.2 (-1.1, 0.8)	0.73	0.09*	129 (-349, 606)	0.59	0.10*

All ΣDAP concentrations are log_{10}-transformed.
[a]Excludes mothers in "other" race category. Adjusted for mother's age, household income, marital status, parity category, log_{10}-transformed blood lead, and log_{10}-transformed cotinine. Models for all mothers are also adjusted for maternal race.
[b]p-value for *PON1* X ΣDAP interaction term from separate cross-product models.
[c]Not enough mothers in category to produce an effect estimate.

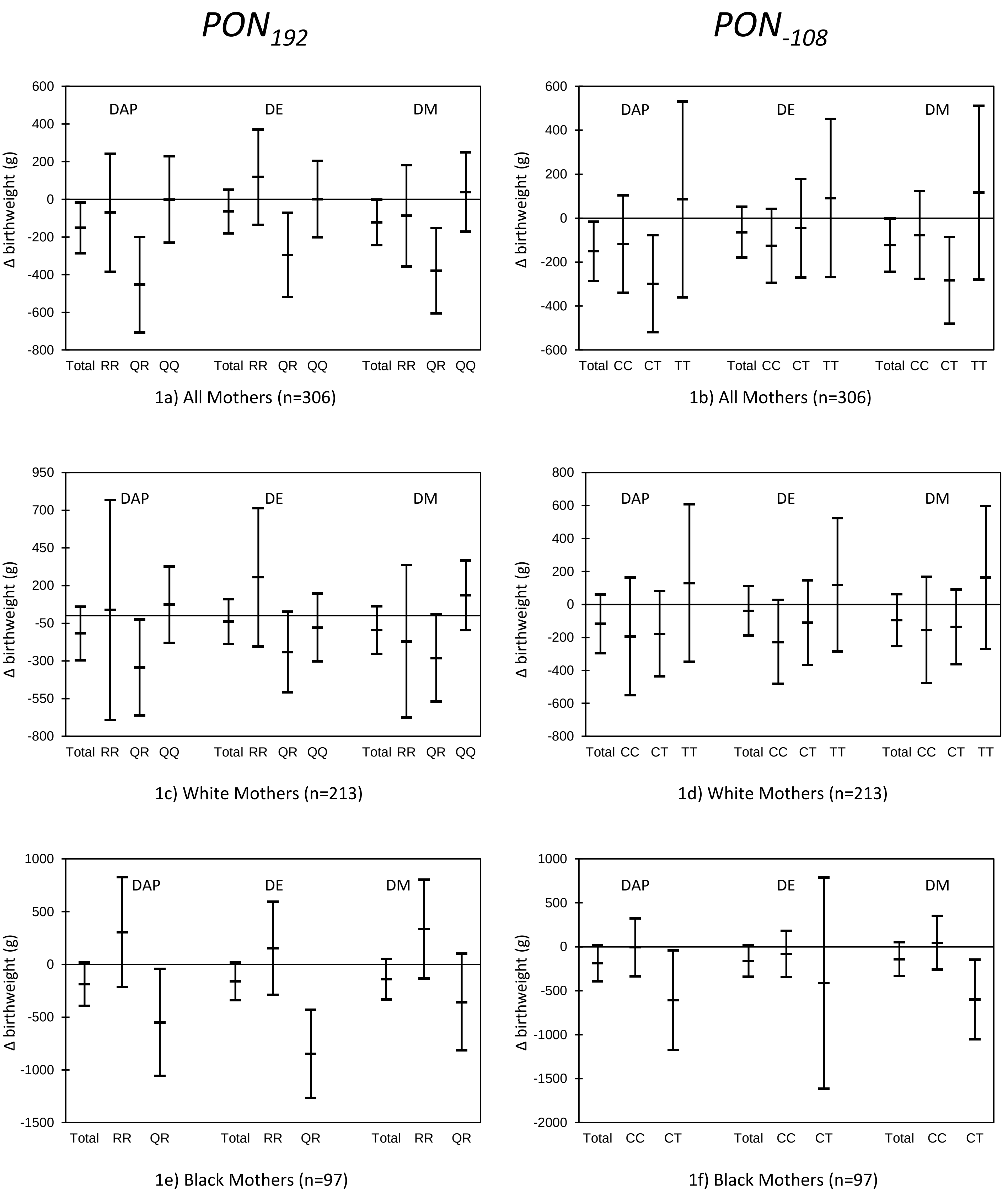

Supplemental Material, Figure 1: *PON1*-stratified associations of a 10-fold increase in ΣDAPs, ΣDEs, and ΣDMs with birthweight, for all mothers (1a and 1b), white mothers (1c and 1d), and black mothers (1e and 1f). All models adjusted for mother's age, household income, marital status, parity category, and $\log_{10}$-transformed blood lead, and $\log_{10}$-transformed cotinine; models for all mothers also adjusted for maternal race. There were not enough infants with black mothers had $PON1_{192QQ}$ (n=7) or $PON1_{-108TT}$ (n=2) genotype to calculate effect estimates. Error bars denote 95% confidence intervals.

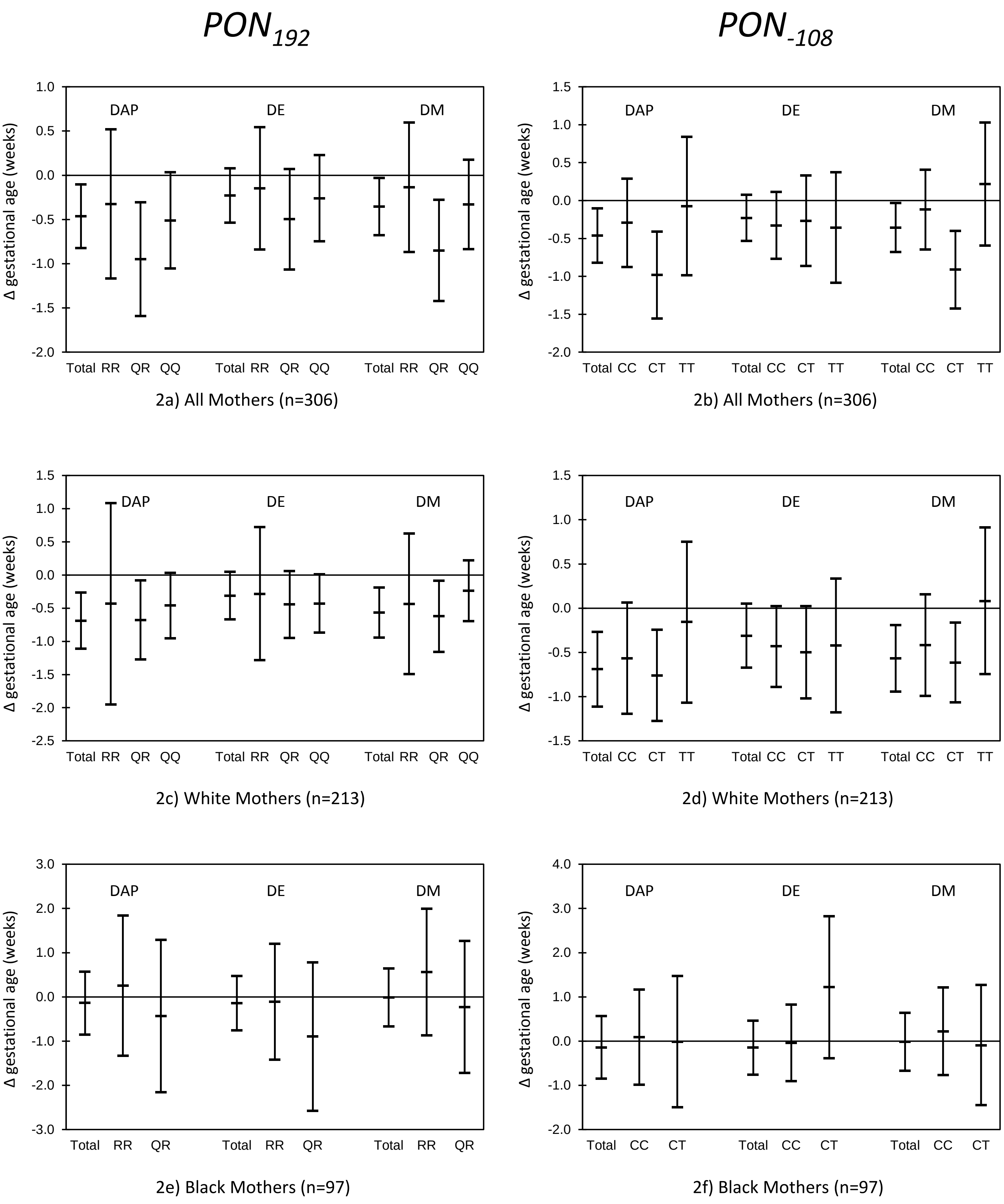

Supplemental Material, Figure 2: *PON1*-stratified associations of a 10-fold increase in ΣDAPs, ΣDEs, and ΣDMs with length of gestation, for all mothers (2a and 2b), white mothers (2c and 2d), and black mothers (2e and 2f). All models adjusted for mother's age, household income, marital status, parity category, and $\log_{10}$-transformed blood lead, and $\log_{10}$-transformed cotinine; models for all mothers also adjusted for maternal race. There were not enough infants with black mothers had $PON1_{192QQ}$ (n=7) or $PON1_{-108TT}$ (n=2) genotype to calculate effect estimates. Error bars denote 95% confidence intervals.
